# Supplementary material for: UV-induced G4 DNA structures recruit ZRF1 which prevents UV-induced senescence
Source: Nat Commun. 2023 Oct 23;14:6705. doi: 10.1038/s41467-023-42494-x (PMC10593929; doi:10.1038/s41467-023-42494-x)
Supplement: Supplementary file 1 — Supplementary Information [file 41467_2023_42494_MOESM1_ESM.pdf]

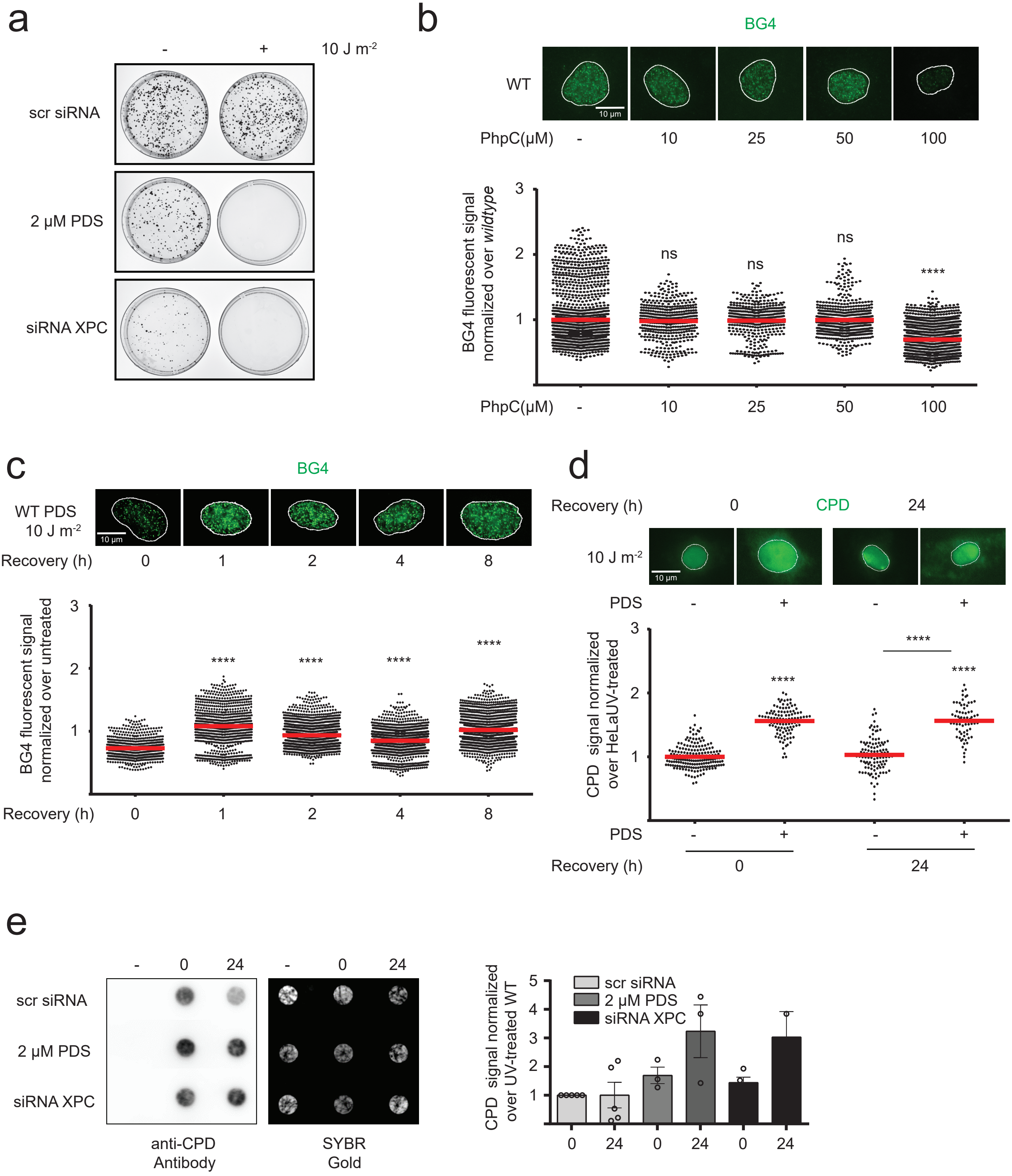

a
- + 10 J m-2
scr siRNA
2 μM PDS
siRNA XPC
b
BG4
WT
10 μm
PhpC(μM) - 10 25 50 100
BG4 fluorescent signal normalized over wildtype
ns ns ns ****
c
BG4
WT PDS 10 J m-2
Recovery (h) 0 1 2 4 8
BG4 fluorescent signal normalized over untreated
d
Recovery (h) 0 CPD 24
10 J m-2
PDS - + - +
CPD signal normalized over HeLaUV-treated
e
- 0 24 - 0 24
scr siRNA
2 μM PDS
siRNA XPC
anti-CPD Antibody
SYBR Gold
CPD signal normalized over UV-treated WT
scr siRNA
2 μM PDS
siRNA XPC
0 24 0 24 0 24

Supplementary Fig. 1 G4s stabilization and the impact on cellular fitness after UV irradiation. a) Colony formation assay in HeLa cells pre-treated/untreated 48 h with 2 μM of PDS or siRNA against XPC, treated/untreated with 10 J m^{-2} UV light and recovered in DMEM/ 10% FBS for 2 weeks. **b)** IF staining of HeLa cells, treated 24 h with different concentrations of the compound PhpC and stained with the BG4 antibody (green) and DAPI (signal was used to indicate the nuclear border as a white line). Scale bar: 10 μm. Below, quantification of BG4 signal in the nucleus of the cells. **c)** IF staining of HeLa cells, pre-treated 48 h with 2 μM of PDS, treated/untreated with 10 J m^{-2} UV light, recovered in DMEM/ 10% FBS for 1 to 8 h and stained with BG4 antibody (green), and DAPI (signal was used to indicate the nuclear border as a white line). Scale bar: 10 μm. Bottom part quantification of n=3 biological independent experiments ± SEM; results were normalized over untreated sample. **d)** IF staining of HeLa cells pre-treated/untreated 48 h with 2 μM of PDS or siRNA against XPC, treated/untreated with 10 J m^{-2} UV light and recovered in DMEM/ 10% FBS for 24 h. The cells were stained with an anti-CPDs antibody (green), and DAPI (signal was used to indicate the nuclear border as a white line). Scale bar: 10 μm. Below, quantification of CPDs signal in the nucleus of the cells. Bottom part quantification of n=3 biological independent experiments ± SEM; results were normalized over HeLa UV-treated cells. Graphs in c) and e) show fluorescence intensity (FI) levels of n=3 biological independent experiments. Horizontal red line represents the mean value. Significance was determined using an ordinary one-sided ANOVA multiple comparison using the Geisser-Greenhouse correction. Asterisks indicate statistical significance; in detail, *$p < 0.05$, **$p < 0.01$, ***$p < 0.001$, ****$p < 0.0001$. Significance compared to untreated-WT cells is indicated by asterisks, connecting lines are used when the significance was compared to other samples. **e)** Dot blot assay of genomic DNA (gDNA) extracted from HeLa cells pre-treated/untreated 48 h with 2 μM of PDS or siRNA against XPC, treated/untreated with 10 J m^{-2} UV light and recovered in DMEM/10% FBS for 24 h. gDNA was spotted on Hybond+ nitrocellulose membrane and stained with an antibody against CPD products. SYBR Gold staining to visualize DNA loading is displayed side by side. The full scan gel image with the raw image is available in Supplementary Fig. 7a (all dot blots of this manuscript were done on a single membrane – see Fig. 3). Bottom part quantification of n=3 biological independent experiments± SEM; results were normalized over HeLa UV-irradiated cells. No significant differences were detected.

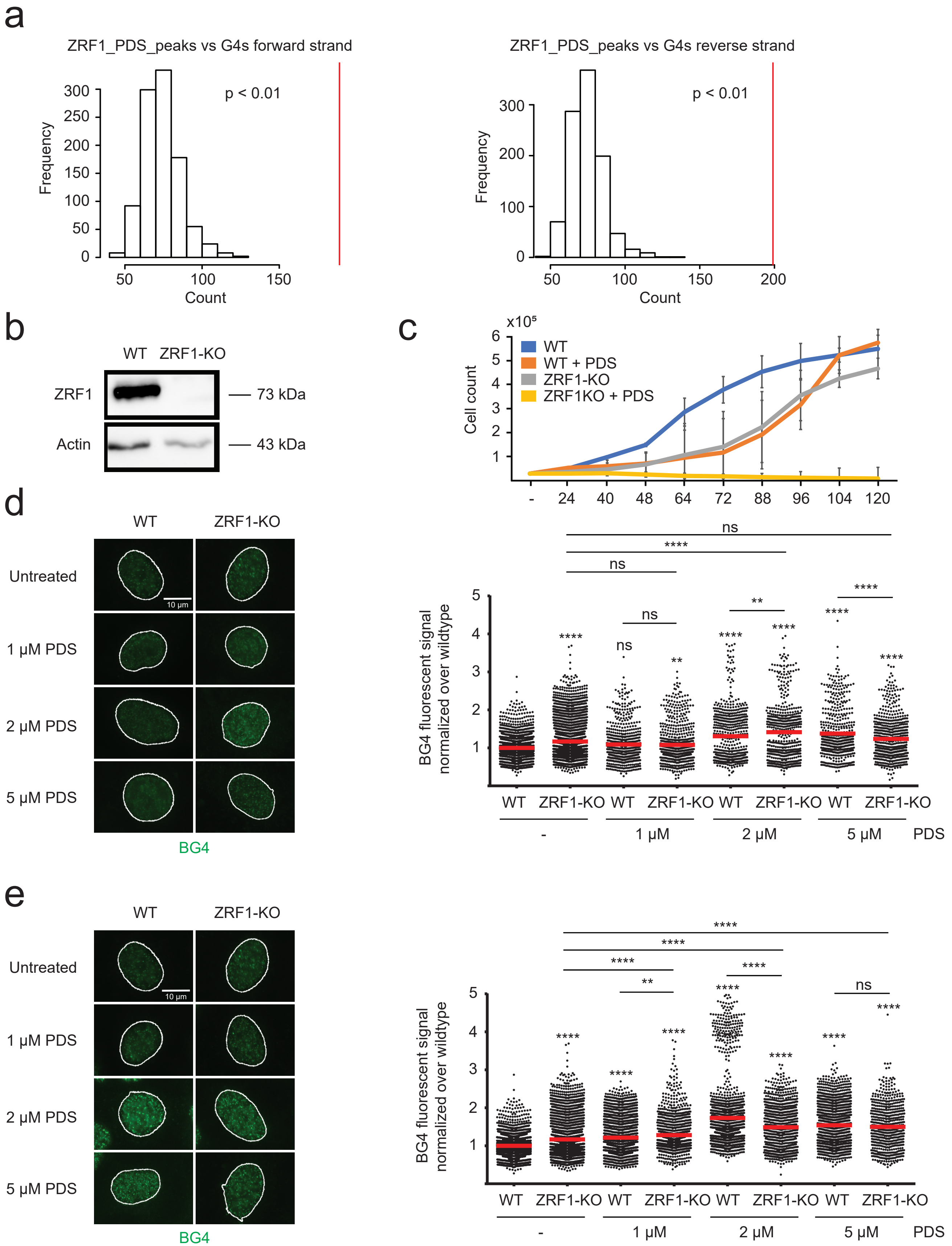

a
ZRF1_PDS_peaks vs G4s forward strand
p < 0.01
Frequency
Count
ZRF1_PDS_peaks vs G4s reverse strand
p < 0.01
b
WT
ZRF1-KO
ZRF1
Actin
73 kDa
43 kDa
c
x10⁵
WT
WT + PDS
ZRF1-KO
ZRF1KO + PDS
Cell count
d
WT
ZRF1-KO
Untreated
1 µM PDS
2 µM PDS
5 µM PDS
10 µm
BG4
BG4 fluorescent signal normalized over wildtype
WT
ZRF1-KO
PDS
1 µM
2 µM
5 µM
ns
****
**
e
WT
ZRF1-KO
Untreated
1 µM PDS
2 µM PDS
5 µM PDS
10 µm
BG4
BG4 fluorescent signal normalized over wildtype
PDS
1 µM
2 µM
5 µM

Supplementary Fig. 2 ZRF1 binds G4s and is involved in attenuating G4 mediated genome instability. a) Genome-wide overlap of the ZRF1 peaks in HeLa cells treated 48 h with 2 µM of PDS with G4 motifs in the forward strand (left) and revers strand (right) as predicted by[14]. The red line indicates a highly significant overlap $p < 0.01$. **b)** Western blot analysis of protein extracts from HeLa WT and CRISPR selected ZRF1-KO cells. Membrane was stained with anti-ZRF1 and anti-actin antibodies. The full scan gel image is reported in Supplementary Fig. 7c. **c)** Growth curves in WT and ZRF1-KO cells treated/untreated 24 h with 10 µM of PDS. Graph shows the mean of n=3 biological independent experiments ± SD. **d-e)** DAPI staining of WT and ZRF1-KO cells, treated/untreated 24 h (d) and 72 h (e) with 1, 2 or 5µM of PDS, stained with BG4 antibody (green), and DAPI (signal was used to indicate the nuclear border as a white line). Scale bar: 10 µm. To the right, quantification of BG4 signal in the nucleus of the cells. Graphs show fluorescence intensity (FI) levels normalized over untreated cells of at least n=3 biological independent experiments. Horizontal red line represents the mean value. Significance was determined using an ordinary one-sided ANOVA multiple comparison using the Geisser-Greenhouse correction. Asterisks indicate statistical significance; in detail, $*p < 0.05$, $**p < 0.01$, $***p < 0.001$, $****p < 0.0001$. Significance compared to untreated-WT cells is indicated by asterisks, connecting lines are used when the significance was compared to other samples.

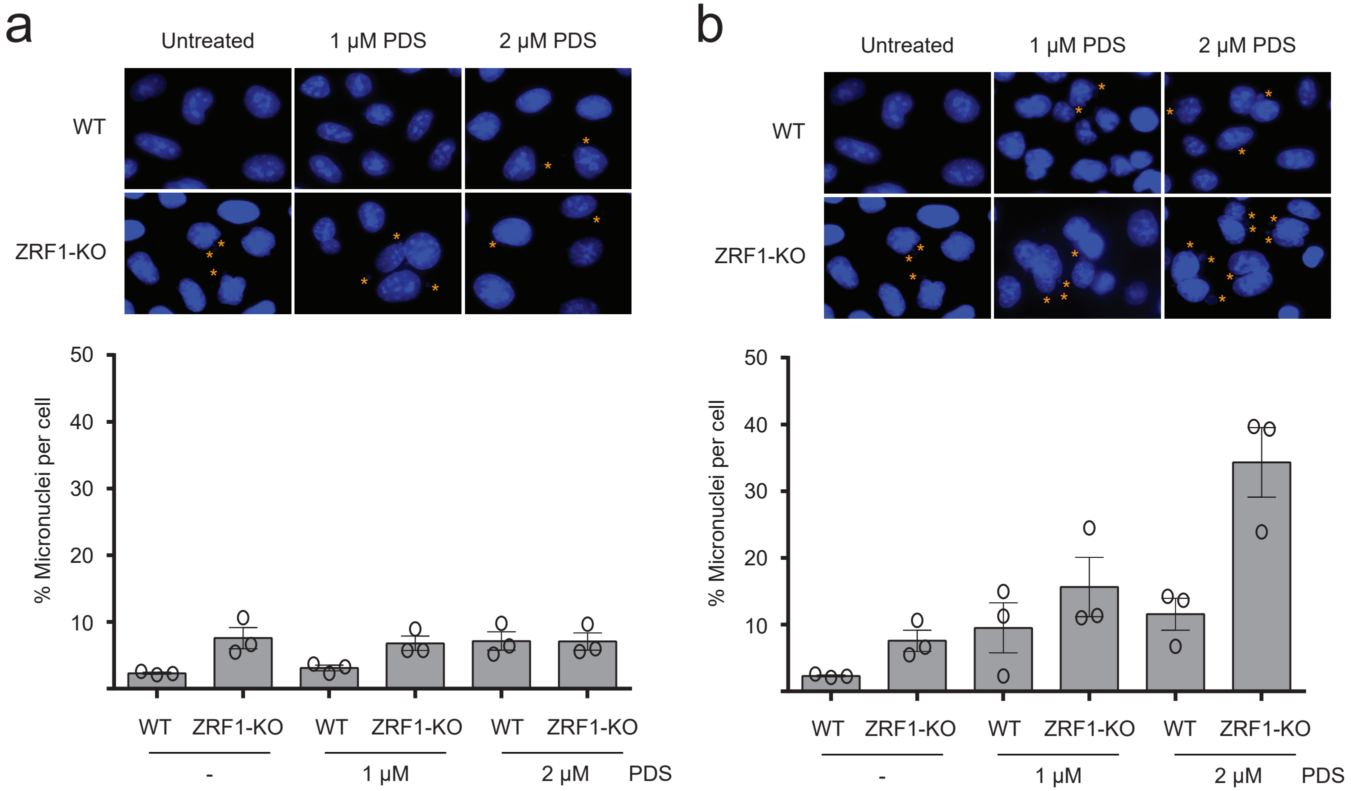
a
Untreated
1 μM PDS
2 μM PDS
WT
ZRF1-KO
% Micronuclei per cell
50
40
30
20
10
WT
ZRF1-KO
WT
ZRF1-KO
WT
ZRF1-KO
-
1 μM
2 μM
PDS
b
Untreated
1 μM PDS
2 μM PDS
WT
ZRF1-KO
% Micronuclei per cell
50
40
30
20
10
WT
ZRF1-KO
WT
ZRF1-KO
WT
ZRF1-KO
-
1 μM
2 μM
PDS

Supplementary Fig. 3 ZRF1 is involved in attenuating G4 mediated micronuclei formation. a-b) DAPI staining of WT and ZRF1-KO cells, treated/untreated 24h (panel a) and 72h (panel b) with 1 or 2 μM of PDS, stained with DAPI (blue). Scale bar: 10 μm. Below, micronuclei quantification as a fraction of number of cells. Bars show mean value of n=3 biological independent experiments ± SEM. Significance compared to untreated-WT cells is indicated by asterisks, connecting lines are used when the significance was compared to other samples.

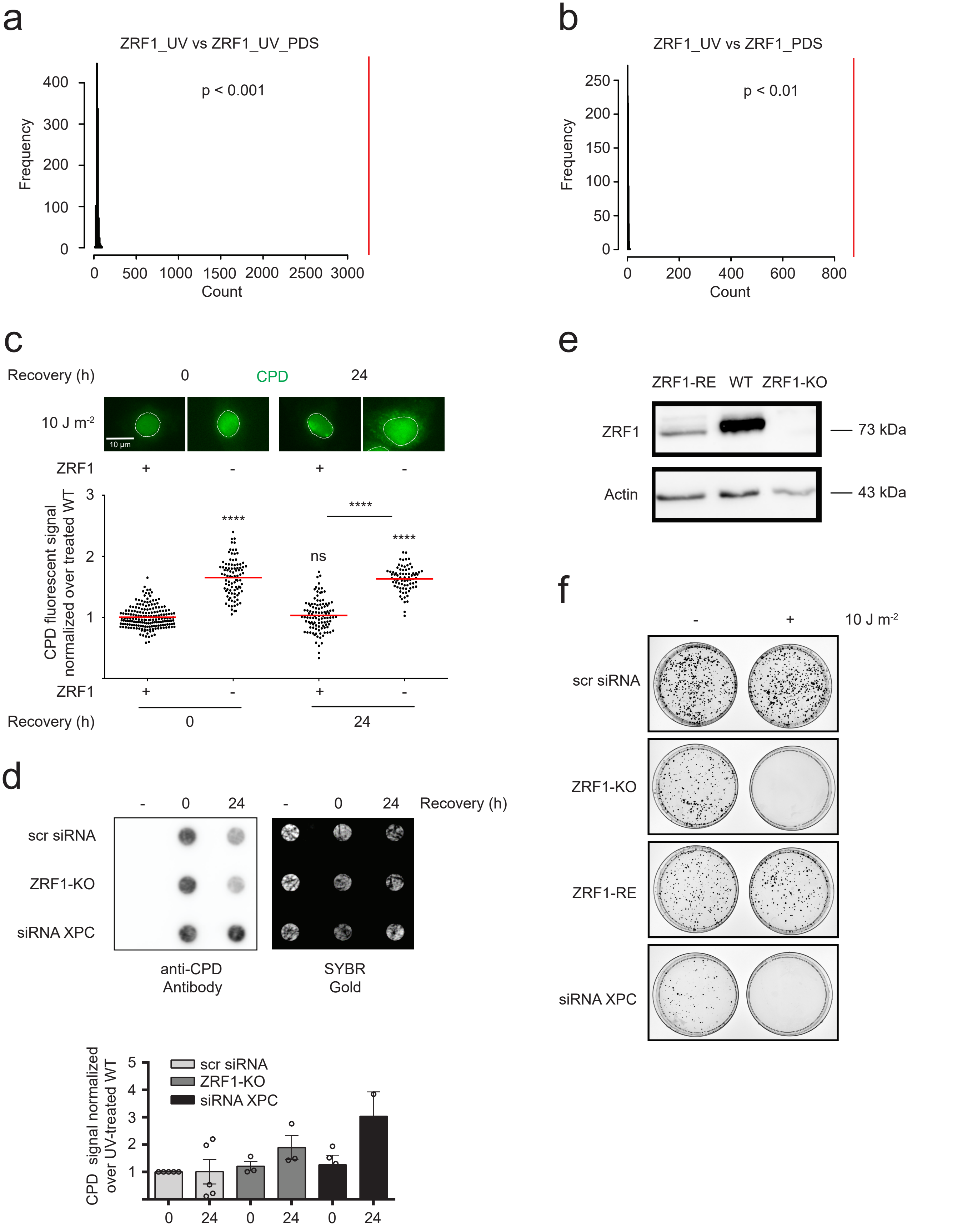

a
ZRF1_UV vs ZRF1_UV_PDS
p < 0.001
Frequency
Count
b
ZRF1_UV vs ZRF1_PDS
p < 0.01
Frequency
Count
c
Recovery (h)
0
CPD
24
10 J m-2
10 µm
ZRF1
CPD fluorescent signal normalized over treated WT
****
ns
d
- 0 24 - 0 24 Recovery (h)
scr siRNA
ZRF1-KO
siRNA XPC
anti-CPD Antibody
SYBR Gold
CPD signal normalized over UV-treated WT
e
ZRF1-RE WT ZRF1-KO
ZRF1
Actin
73 kDa
43 kDa
f
- + 10 J m-2
scr siRNA
ZRF1-KO
ZRF1-RE
siRNA XPC

Supplementary Fig. 4 ZRF1 activity is evolutionary conserved but with an opposite phenotype compared to Zuo1.

a) Genome-wide overlap of the ZRF1 peaks in HeLa cells treated with 10 J m^{-2} UV light with the peaks obtained in cells pre-treated 48 h with 2 μM of PDS and treated with 10 J m^{-2} UV light. The red line indicates a highly significant overlap $p < 0.001$. **b)** Genome-wide overlap of the ZRF1 peaks in HeLa cells treated with 10 J m^{-2} UV light with the peaks obtained in cells treated 48 h with 2 μM of PDS. The red line indicates a highly significant overlap $p < 0.01$. **c)** IF staining of WT and ZRF1-KO treated/untreated with 10 J m^{-2} UV light and recovered in DMEM/10% FBS for 24 h. The cells were stained with an anti-CPDs antibody (green), and DAPI (nucleus border is defined by white borders). Scale bar: 10 μm. Below, quantification of CPDs signal in the nucleus of the cells. Bottom part quantification of n=3 biological independent experiments ± SEM. Graph show fluorescence intensity (FI) levels normalized over WT treated cells of n=3 biological independent experiments. Horizontal red line represents the mean value. Significance was determined using an ordinary one-sided ANOVA multiple comparison using the Geisser-Greenhouse correction. Asterisks indicate statistical significance; in detail, *$p < 0.05$, **$p < 0.01$, ***$p < 0.001$, ****$p < 0.0001$. **d)** Dot blot assay of genomic DNA (gDNA) extracted from WT and ZRF1-KO as well as WT transfected with a siRNA against XPC, treated/untreated with 10 J m^{-2} UV light and recovered in DMEM/10% FBS for 24 h. gDNA was spotted on Hybond+ nitrocellulose membrane and stained with an antibody against CPD products. SYBR Gold staining to visualize DNA loading is displayed side by side. Note, the presented dot blot of WT is the same as in Supplementary Fig. 1 as all dot blots were perform on one membrane. The full scan gel image of the dot blot presented in Supplementary Fig. 1 and Supplementary Fig. 4 is reported in Supplementary Fig. 7a. Bottom part, quantification of n=3 biological independent experiments, results were normalized over HeLa UV-treated cells. No significant differences were detected. **e)** Western blot analysis of protein extracts from HeLa WT and ZRF1-KO cells transfected/untransfected with ZRF1 plasmid. Membrane was stained with anti-ZRF1 and anti-Actin antibodies. The full scan gel image is reported in Supplementary Fig. 7d. Significance compared to untreated-WT cells is indicated by asterisks, connecting lines are used when the significance was compared to other samples. **f)** Colony formation assay in WT and ZRF1-KO cells. WT cells were transfected with a siRNA against XPC, as well as ZRF1-KO cells with constitutive ZRF1 exogenous protein expression (ZRF1-RE) and treated/untreated with 10 J m^{-2} UV light and recovered in DMEM/10% FBS

for 2 weeks. The colony formation experiments are part of the same experiment shown in Supplementary Fig. 1a.

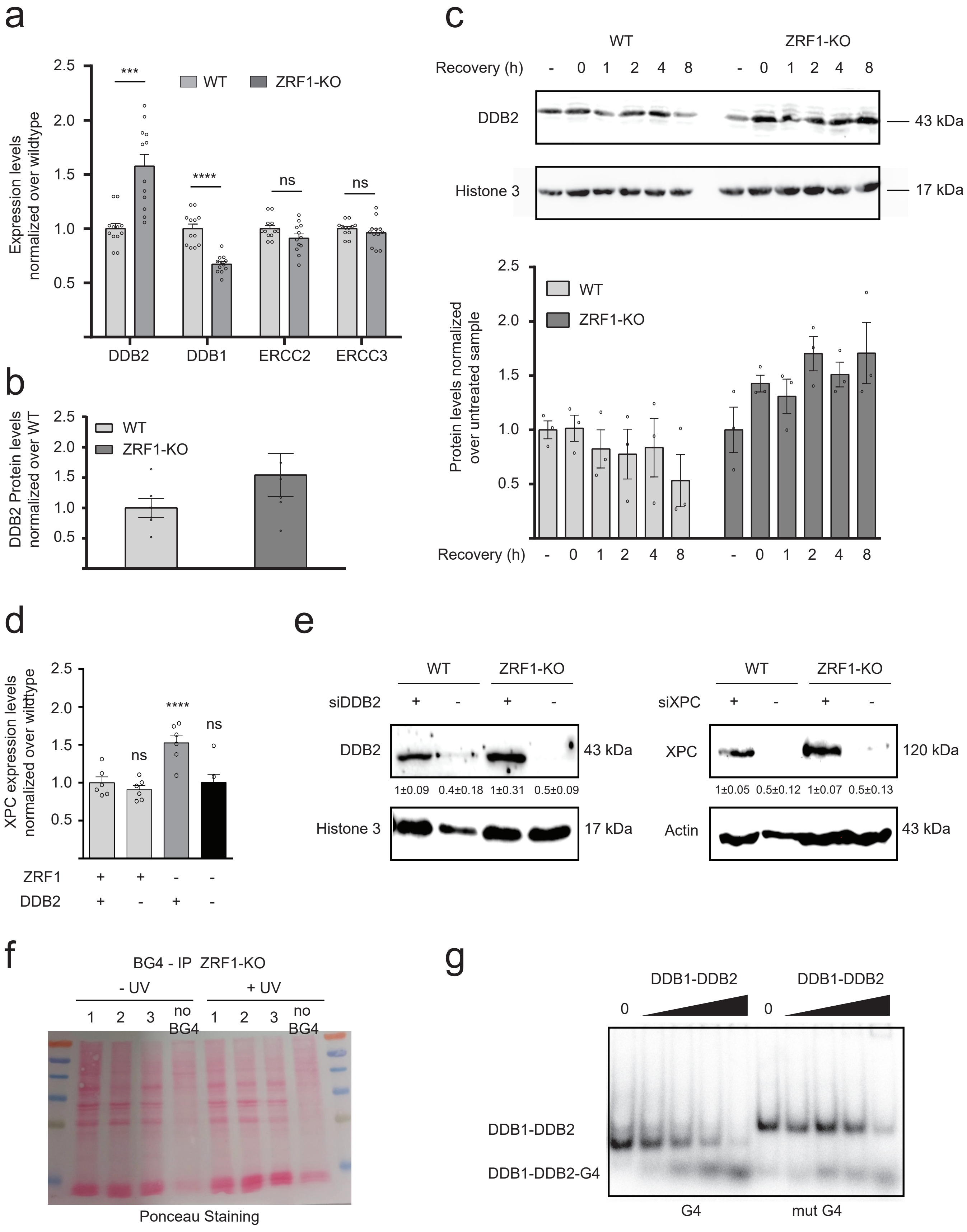
a
Expression levels normalized over wildtype
WT
ZRF1-KO
***
****
ns
ns
DDB2
DDB1
ERCC2
ERCC3
b
DDB2 Protein levels normalized over WT
WT
ZRF1-KO
c
WT
ZRF1-KO
Recovery (h)
DDB2
43 kDa
Histone 3
17 kDa
Protein levels normalized over untreated sample
WT
ZRF1-KO
Recovery (h)
d
XPC expression levels normalized over wildtype
****
ns
ns
ZRF1
DDB2
e
WT
ZRF1-KO
siDDB2
DDB2
43 kDa
1±0.09
0.4±0.18
1±0.31
0.5±0.09
Histone 3
17 kDa
WT
ZRF1-KO
siXPC
XPC
120 kDa
1±0.05
0.5±0.12
1±0.07
0.5±0.13
Actin
43 kDa
f
BG4 - IP ZRF1-KO
- UV
+ UV
no BG4
no BG4
Ponceau Staining
g
DDB1-DDB2
DDB1-DDB2
DDB1-DDB2
DDB1-DDB2-G4
G4
mut G4

Supplementary Fig. 5 DDB2 upregulation mediated NER activity at G4.

a). Expression levels of selected up and downregulated genes. mRNA levels were normalized to the level of U6 snRNA and GAPDH. WT mRNA levels were scaled to 1. Error bars represent SEM of 12 biological independent experiments. **b)** Quantification of western blot analysis of protein extracts from wildtype and ZRF1-KO cells. No significant differences were detected. **c)** Western blot analysis of protein extracts from wildtype and ZRF1-KO cells untreated or treated with 10 J m^{-2} UV light and recovered from 0 to 8 h in DMEM/10% FBS. Membrane was stained with anti-DDB2 and anti-histone 3 antibodies. No significant differences were detected. The full scan gel image is reported in Supplementary Fig. 7e. **d)** BG4 immunoprecipitation of ZRF1-KO cells untreated or treated with 10 J m^{-2} UV light and stained with Ponceau. **e)** 100 nM radiolabelled ssDNA (30 nt) was incubated at increasing concentrations of DDB1-DDB2 heterodimer protein, indicated by the black triangle 0, 12.5, 25, 50, 100 nM). The complexes were resolved on a 6% native polyacrylamide (19:1) gel **f)** Expression levels of XPC transcript in WT and ZRF1-KO transfected with siRNA against DDB2. mRNA levels were normalized to the level of U6 snRNA and GAPDH. WT mRNA levels were scaled to 1. Error bars represent SEM of at least n=3 biological independent experiments. **g)** Western blot analysis of protein extracts from WT and ZRF1-KO cells transfected/untransfected 48 h with DDB2 (left) and XPC (right) siRNA and/or recovered 24 h in DMEM/10% FBS. Membrane was stained with anti-DDB2 or anti-XPC and anti-histone 3 or anti-Actin antibodies. The full scan gel image is reported in Supplementary Fig. 7f.

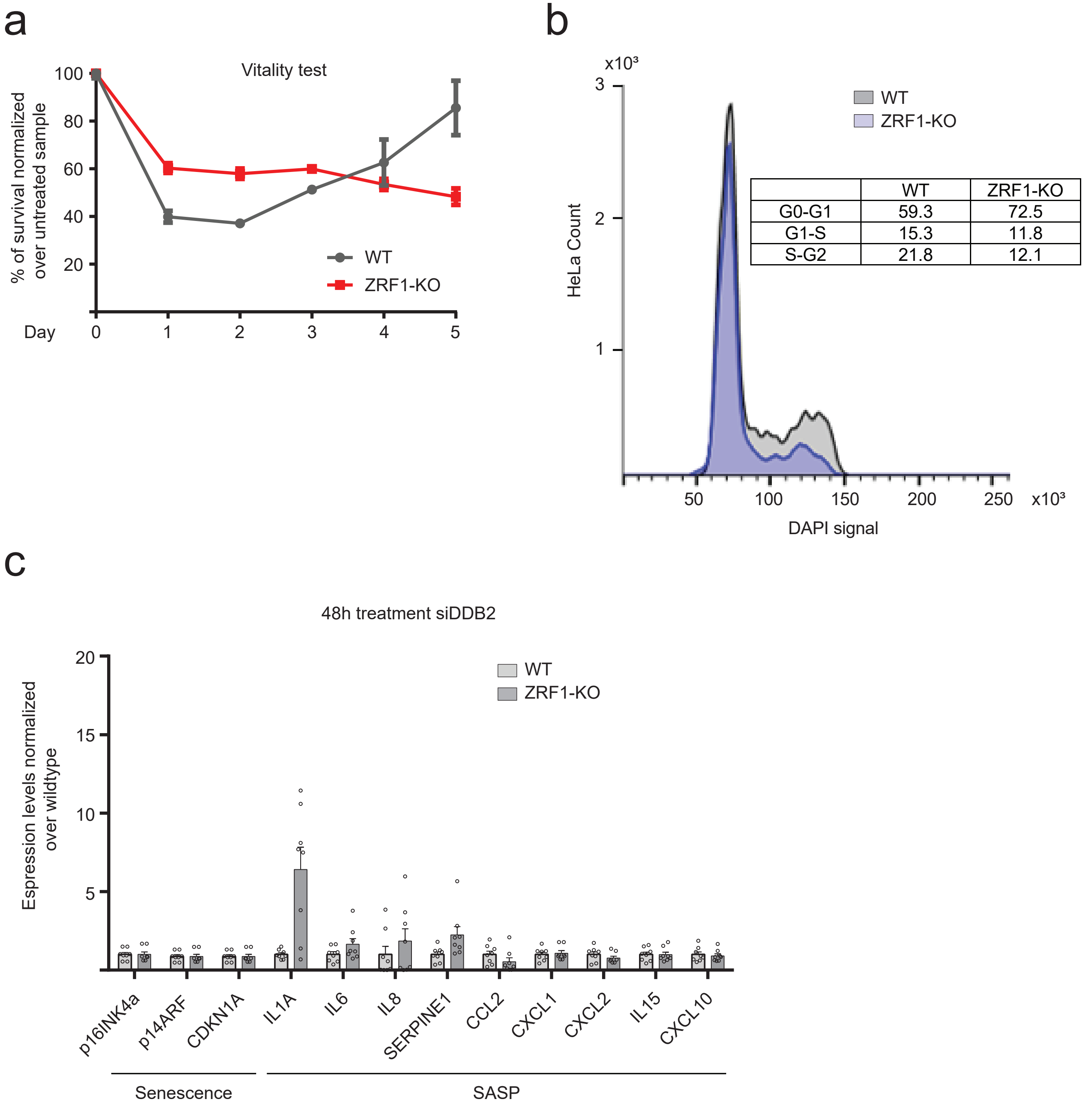
a
Vitality test
% of survival normalized over untreated sample
100
80
60
40
20
Day
0
1
2
3
4
5
WT
ZRF1-KO
b
x10³
3
2
1
HeLa Count
WT
ZRF1-KO
	WT	ZRF1-KO
G0-G1	59.3	72.5
G1-S	15.3	11.8
S-G2	21.8	12.1
50
100
150
200
250
x10³
DAPI signal
c
48h treatment siDDB2
Espression levels normalized over wildtype
20
15
10
5
WT
ZRF1-KO
p16INK4a
p14ARF
CDKN1A
IL1A
IL6
IL8
SERPINE1
CCL2
CXCL1
CXCL2
IL15
CXCL10
Senescence
SASP

Supplementary Fig. 6 ZRF1 prevents CDKN2A upregulation and UV-induced senescence. **a)** Growth curves in WT (grey line) and ZRF1-KO (red line) cells treated with 10 J m^{-2} UV light and recovered for 1 to 5 days in DMEM/10% FBS. Graph shows mean of n=3 biological independent experiments ± SD. **b)** Histogram plot of the DAPI signal in WT and ZRF1-KO cells, treated with 10 J m^{-2} UV light and recovered 5 days in DMEM/10% FBS. The cell cycle distribution was obtained dividing the cells per DAPI amount. The table states the quantification (% of total) of the cells in the three cell cycle phases. **c)** Expression levels of marker genes of senescence in WT and ZRF1-KO cells transfected with siRNA against DDB2. mRNA levels were normalized to the level of U6 snRNA and GAPDH. WT mRNA levels were scaled to 1. Error bars represent SEM of at least n=3 biological independent experiments.

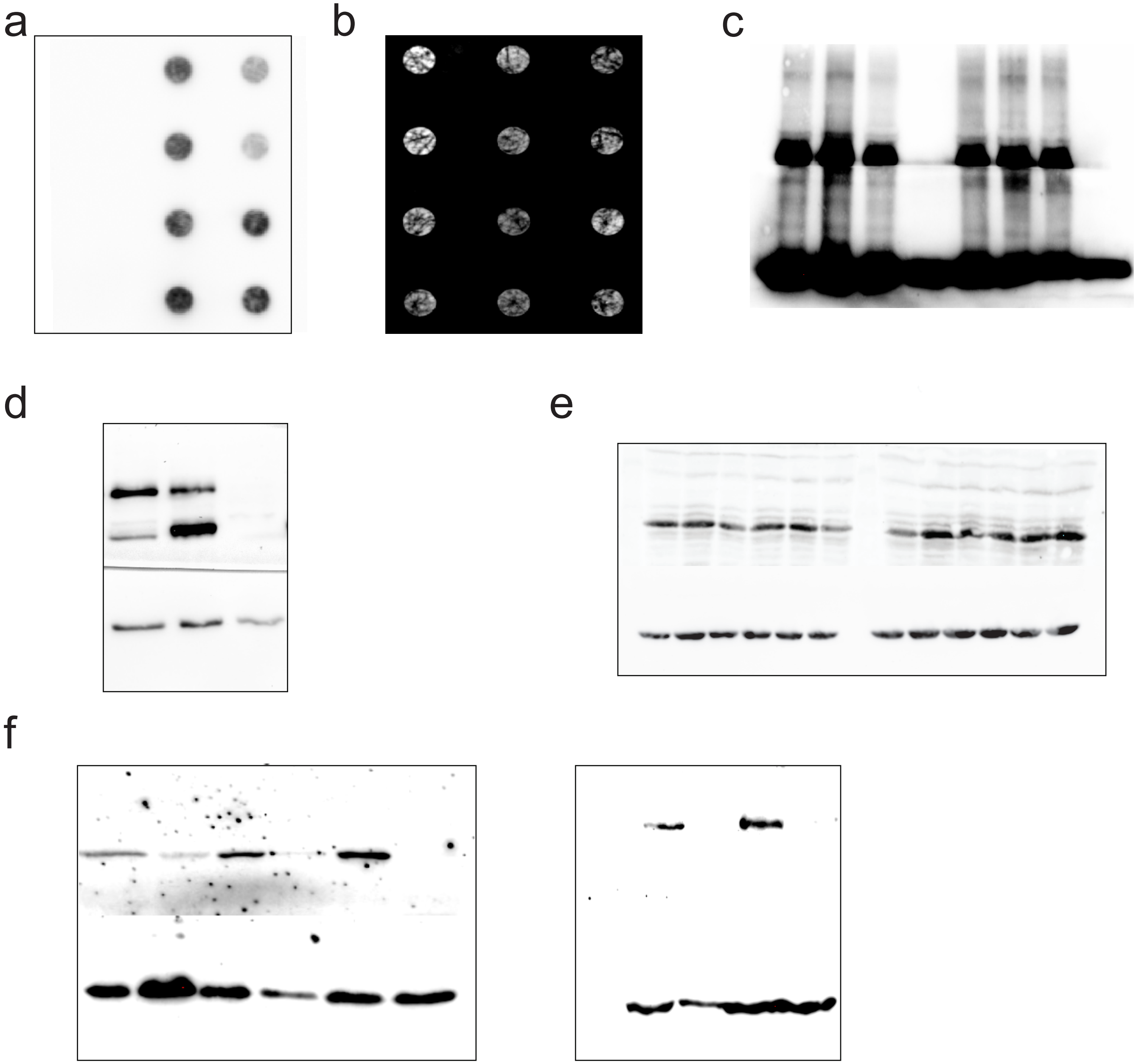
a
b
c
d
e
f

Supplementary Fig. 7 Full scan images.

a) Full scan gel image of Fig. 1d and 3e. **b)** Full scan gel image of Supplementary Fig. 1d and 4c. **c)** Full scan gel image of Fig. 4d. **d)** Full scan gel image of Supplementary Fig. 4e. **e)** Full scan gel image of Supplementary Fig. 5b. **f)** Full scan gel image of Supplementary Fig. 5e.

TABLES

Supplementary Table 1. DEGs involved in cellular senescence upregulated in UV conditions.

Gene symb	Total coun	P-value (KO UV vs. WT UV)	FDR step u	Ratio (KO U	Fold chang	LSMean(KO	LSMean(W
CDKN2A	3,02E+03	1,28E-08	1,73E-06	1,47E+00	1,47E+00	5,40E+02	3,68E+02
CDK6	1,93E+03	1,25E-05	9,79E-04	2,24E+00	2,24E+00	9,50E+01	4,24E+01
B2M	1,70E+04	3,07E-05	2,13E-03	8,84E-01	-1,13E+00	2,17E+03	2,45E+03
CDKN1A	6,01E+02	3,54E-03	1,03E-01	1,57E+00	1,57E+00	1,08E+02	6,88E+01
NUAK1	1,75E+02	4,31E-03	1,19E-01	5,63E+00	5,63E+00	1,81E+01	3,21E+00
MAP2K3	7,36E+02	6,16E-03	1,52E-01	1,70E+00	1,70E+00	7,17E+01	4,21E+01
MME	5,95E+01	2,41E-02	3,98E-01	6,57E+00	6,57E+00	1,09E+01	1,65E+00
ROMO1	3,19E+03	2,82E-02	4,37E-01	1,16E+00	1,16E+00	4,96E+02	4,29E+02
MAP2K6	2,04E+02	2,92E-02	4,45E-01	1,83E+00	1,83E+00	3,71E+01	2,03E+01
IGF1R	3,19E+02	3,16E-02	4,66E-01	2,01E+00	2,01E+00	2,87E+01	1,43E+01

T UV) (KO UV vs. WT UV)
