# Supplementary material for: UV-induced G4 DNA structures recruit ZRF1 which prevents UV-induced senescence
Source: Nat Commun. 2023 Oct 23;14:6705. doi: 10.1038/s41467-023-42494-x (PMC10593929; doi:10.1038/s41467-023-42494-x)
Supplement: Supplementary file 3 — Description of Additional Supplementary Files [file 41467_2023_42494_MOESM3_ESM.pdf]

Description of Additional Supplementary Files

File Name: Supplementary Data 1
Description: ZRF1 peaks detected by ChIPseq after PDS treatment

File Name: Supplementary Data 2
Description: ZRF1 peaks detected by ChIPseq after UV treatment

File Name: Supplementary Data 3
Description: ZRF1 peaks detected by ChIPseq after PDS and UV treatment

File Name: Supplementary Data 4
Description: 3' RNA-seq with both WT and ZRF1-KO cells. List contains differently expressed genes (DEGs)

File Name: Supplementary Data 5
Description: 3' RNA-seq with both WT and ZRF1-KO cells after UV. List contains differently expressed genes (DEGs)

File Name: Supplementary Data 6
Description: Unique DEGS that were only present after UV treatment
