# Supplementary material for: UV-induced G4 DNA structures recruit ZRF1 which prevents UV-induced senescence
Source: Nat Commun. 2023 Oct 23;14:6705. doi: 10.1038/s41467-023-42494-x (PMC10593929; doi:10.1038/s41467-023-42494-x)
Supplement: Supplementary file 10 — Reporting Summary [file 41467_2023_42494_MOESM10_ESM.pdf]

nature portfolio

Corresponding author(s): Katrin Paeschke

Last updated by author(s): Sep 20, 2023

Reporting Summary

Nature Portfolio wishes to improve the reproducibility of the work that we publish. This form provides structure for consistency and transparency in reporting. For further information on Nature Portfolio policies, see our Editorial Policies and the Editorial Policy Checklist.

Statistics

For all statistical analyses, confirm that the following items are present in the figure legend, table legend, main text, or Methods section.

n/a	Confirmed	
☐	☒	The exact sample size (*n*) for each experimental group/condition, given as a discrete number and unit of measurement
☐	☒	A statement on whether measurements were taken from distinct samples or whether the same sample was measured repeatedly
☐	☒	The statistical test(s) used AND whether they are one- or two-sided<br>*Only common tests should be described solely by name; describe more complex techniques in the Methods section.*
☐	☒	A description of all covariates tested
☐	☒	A description of any assumptions or corrections, such as tests of normality and adjustment for multiple comparisons
☐	☒	A full description of the statistical parameters including central tendency (e.g. means) or other basic estimates (e.g. regression coefficient) AND variation (e.g. standard deviation) or associated estimates of uncertainty (e.g. confidence intervals)
☐	☒	For null hypothesis testing, the test statistic (e.g. *F*, *t*, *r*) with confidence intervals, effect sizes, degrees of freedom and *P* value noted<br>*Give P values as exact values whenever suitable.*
☒	☐	For Bayesian analysis, information on the choice of priors and Markov chain Monte Carlo settings
☒	☐	For hierarchical and complex designs, identification of the appropriate level for tests and full reporting of outcomes
☒	☐	Estimates of effect sizes (e.g. Cohen's *d*, Pearson's *r*), indicating how they were calculated

Our web collection on statistics for biologists contains articles on many of the points above.

Software and code

Policy information about availability of computer code

Data collection	No software was used
Data analysis	Microsoft Excel (latest release), ImageJ (latest release), GraphPad Prism 6

For manuscripts utilizing custom algorithms or software that are central to the research but not yet described in published literature, software must be made available to editors and reviewers. We strongly encourage code deposition in a community repository (e.g. GitHub). See the Nature Portfolio guidelines for submitting code & software for further information.

Data

Policy information about availability of data

All manuscripts must include a data availability statement. This statement should provide the following information, where applicable:

- Accession codes, unique identifiers, or web links for publicly available datasets
- A description of any restrictions on data availability
- For clinical datasets or third party data, please ensure that the statement adheres to our policy

ChIP-seq data have been uploaded in the National Center for Biotechnology Information (NCBI) Sequencing Read Archive under the reference number PRJNA817435. All data is available upon request from the corresponding author.

Human research participants

Policy information about studies involving human research participants and Sex and Gender in Research.

Reporting on sex and gender	N/A
Population characteristics	N/A
Recruitment	N/A
Ethics oversight	N/A

Note that full information on the approval of the study protocol must also be provided in the manuscript.

Field-specific reporting

Please select the one below that is the best fit for your research. If you are not sure, read the appropriate sections before making your selection.

☒ Life sciences ☐ Behavioural & social sciences ☐ Ecological, evolutionary & environmental sciences

For a reference copy of the document with all sections, see nature.com/documents/nr-reporting-summary-flat.pdf

Life sciences study design

All studies must disclose on these points even when the disclosure is negative.

Sample size	Plotted results were based on the average of, at least, N=3 biologically independent experiments. Significance was calculated using one-sided Student's t-test and ordinary one-way ANOVA multiple comparison tests.
Data exclusions	No data were excluded from the analyses
Replication	All the biologically independent replication (3 or more) were succesfull
Randomization	Randomization was not applicable to our study.
Blinding	Blinding was not relevant for our study because we had no idea about the outcome of the single experiments. Some of the results were against the previously published trend in S.Cerevisiae. We shed light which was the reason why we had this different behaviour in Human.

Reporting for specific materials, systems and methods

We require information from authors about some types of materials, experimental systems and methods used in many studies. Here, indicate whether each material, system or method listed is relevant to your study. If you are not sure if a list item applies to your research, read the appropriate section before selecting a response.

Materials & experimental systems

n/a	Involved in the study	
☐	☒	Antibodies
☐	☒	Eukaryotic cell lines
☒	☐	Palaeontology and archaeology
☒	☐	Animals and other organisms
☒	☐	Clinical data
☒	☐	Dual use research of concern

Methods

n/a	Involved in the study	
☐	☒	ChIP-seq
☐	☒	Flow cytometry
☒	☐	MRI-based neuroimaging

Antibodies

Antibodies used	Antibody against CPD: Anti-Thymine Dimer mAb, clone KTM53 (Kamiya Biomedical Company ref# MC-062)<br>Antibody against 6-4 PPs: (Cosmo Bio LTD - Clone 64M-2)<br>BG4 antibody (homemade expression)<br>Anti-Flag antibody (Cell Signalling ref #2368)<br>HRP-coupled anti-rabbit secondary antibody (Santa Cruz sc-2357)<br>HRP-coupled anti-mouse secondary antibody (Santa Cruz sc-516102)

	Anti-ZRF1 antibody (Novus Biologicals ref #NBP2-12802)<br>Anti-DDB2 antibody (Santa Cruz sc-81246)<br>Anti-XPC antibody (Santa Cruz sc-74410)<br>Anti-rabbit IgG (Life technologies ref #A10520)<br>Anti-phospho-Histone H2A.X (Ser139) Antibody, clone JBW301 (Merck ref #05-636)
Validation	BG4 antibody was monitored by SDS-PAGE and ELISA after each preparation.<br>Anti-CPD antibody checked by dot blot after UV irradiation.<br>Anti-6-4 PPs antibody checked by immunofluorescence after UV radiation.<br>ZRF1, DDB2, XPC, H2A.X antibodies by Western Blot.

Eukaryotic cell lines

Policy information about cell lines and Sex and Gender in Research

Cell line source(s)	HeLa T-Rex Flp-In cells were purchased from Thermo Fischer Scientific
Authentication	None of the cell lines used were autenthicated
Mycoplasma contamination	All the cell lines tested negative for Mycoplasma contamination
Commonly misidentified lines (See ICLAC register)	No commonly misidentified cell lines were used.

ChIP-seq

Data deposition

☒ Confirm that both raw and final processed data have been deposited in a public database such as GEO.

☒ Confirm that you have deposited or provided access to graph files (e.g. BED files) for the called peaks.

Data access links<br>*May remain private before publication.*	https://submit.ncbi.nlm.nih.gov/subs/sra/SUB11197185/overview
Files in database submission	Bam files of the ChIP-seq experiment and file with the peaks calles by MACS2
Genome browser session (e.g. UCSC)	*Provide a link to an anonymized genome browser session for "Initial submission" and "Revised version" documents only, to enable peer review. Write "no longer applicable" for "Final submission" documents.*

Methodology

Replicates	ChIP-seq was performed as a duplicate
Sequencing depth	ZRF1_WT1.fq 38297047 reads; of these: 38297047 (100.00%) were paired.<br>ZRF1_WT2.fq 34600795 reads; of these: 34600795 (100.00%) were paired.<br>ZRF1_KO1.fq 35025083 reads; of these: 35025083 (100.00%) were paired.<br>ZRF1_Input1.fq 19616075 reads; of these: 19616075 (100.00%) were paired.<br>ZRF1_UV1.fq 35358700 reads; of these: 35358700 (100.00%) were paired.<br>ZRF1_UV2.fq 34038777 reads; of these: 34038777 (100.00%) were paired;.<br>ZRF1_PDS1.fq 26709551 reads; of these: 26709551 (100.00%) were paired<br>ZRF1_PDS2.fq 33750507 reads; of these: 33750507 (100.00%) were paired<br>ZRF1_UV_PDS1.fq 34083131 reads; of these: 34083131 (100.00%) were paired.<br>ZRF1_UV_PDS2.fq 45915730 reads; of these:45915730 (100.00%) were paired.
Antibodies	Anti-ZRF1 antibody (Novus Biologicals ref #NBP2-12802)
Peak calling parameters	Peaks were called over a control file ZRF1_Input1.fq<br>The settings used for the calling were :<br>lower mfold bound: 5<br>upper mfold bound: 50<br>Band width for picking regions to compute fragment size: 300<br>Peak detection based on: qvalue<br>Minimum FDR (q-value) cutoff for peak detection: 0.05
Data quality	Fastq files were controlled by fastqc.
Software	MACS2, Bowtie2 and fastQC was used

Flow Cytometry

Plots

Confirm that:

☒ The axis labels state the marker and fluorochrome used (e.g. CD4-FITC).

☒ The axis scales are clearly visible. Include numbers along axes only for bottom left plot of group (a 'group' is an analysis of identical markers).

☒ All plots are contour plots with outliers or pseudocolor plots.

☒ A numerical value for number of cells or percentage (with statistics) is provided.

Methodology

Sample preparation	HeLa T-Rex Flp-In cells, wildtype and ZRF1-KO, treated or untreated with UV, were staining with DAPI as performed in previously publications.
Instrument	BD FACSCanto™ II Cell Analyzer
Software	After data acquisition, data was analyzed using FlowJo.
Cell population abundance	All the cells were positive to DAPI staining.
Gating strategy	The cell were gated for the size (forward scatter (FSC)) and granularity of the cells (side scatter (SSC)).

☐ Tick this box to confirm that a figure exemplifying the gating strategy is provided in the Supplementary Information.
