# Supplementary material for: UV-induced G4 DNA structures recruit ZRF1 which prevents UV-induced senescence
Source: Nat Commun. 2023 Oct 23;14:6705. doi: 10.1038/s41467-023-42494-x (PMC10593929; doi:10.1038/s41467-023-42494-x)
Supplement: Supplementary file 11 — Source Data [file 41467_2023_42494_MOESM11_ESM.pdf]

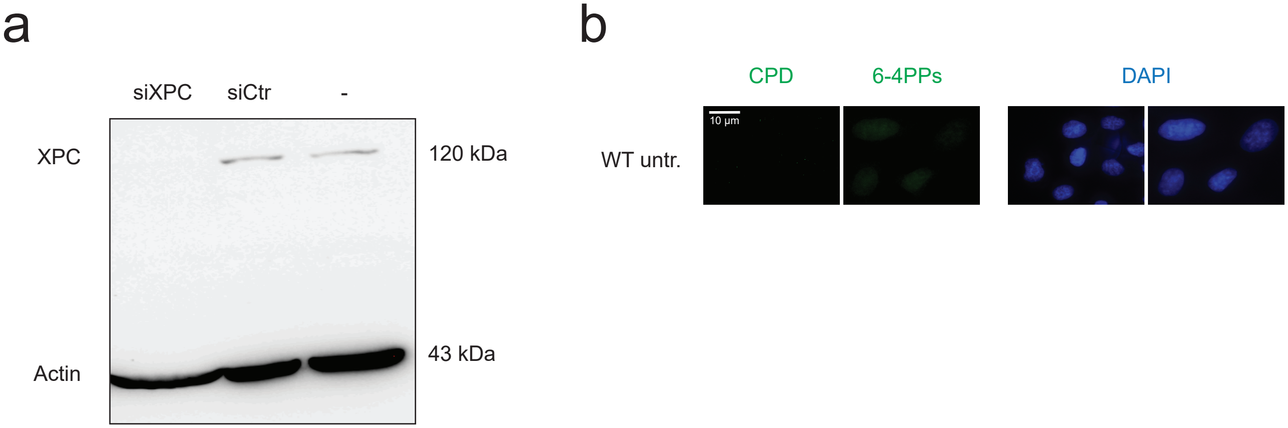

Fig. 1 a) Western blot analysis of protein extracts from WT untrasfected (third lane) or transfected 48 h with siRNA against XPC (first lane) or siRNA ctr (second lane). Membrane was stained with anti-XPC and anti-Actin antibodies. b) IF staining of untreated WT cells stained with an anti-CPDs antibody (left) and anti-6-4PPs antibody (right), and DAPI. Scale bar: 10 µm
